# Preferential expression of functional IL-17R in glioma stem cells: potential role in self-renewal

**DOI:** 10.18632/oncotarget.6847

**Published:** 2016-01-08

**Authors:** Prahlad Parajuli, Rohit Anand, Chandramouli Mandalaparty, Raviteja Suryadevara, Preethi U. Sriranga, Sharon K. Michelhaugh, Simona Cazacu, Susan Finniss, Archana Thakur, Lawrence G. Lum, Dana Schalk, Chaya Brodie, Sandeep Mittal

**Affiliations:** ^1^ Department of Neurosurgery, Wayne State University and Karmanos Cancer Institute, Detroit, MI, USA; ^2^ Department of Oncology, Wayne State University and Karmanos Cancer Institute, Detroit, MI, USA; ^3^ Departments of Internal Medicine, Immunology and Microbiology, and Pediatrics, Wayne State University, Detroit, MI, USA; ^4^ Hermelin Brain Tumor Center, Henry Ford Hospital, Detroit, MI, USA

**Keywords:** glioma stem cells (GSCs), IL-17, IL-17R, IL-6, NF-κB

## Abstract

Gliomas are the most common primary brain tumor and one of the most lethal solid tumors. Mechanistic studies into identification of novel biomarkers are needed to develop new therapeutic strategies for this deadly disease. The objective for this study was to explore the potential direct impact of IL-17−IL-17R interaction in gliomas. Immunohistochemistry and flow cytometry analysis of 12 tumor samples obtained from patients with high grade gliomas revealed that a considerable population (2–19%) of cells in all malignant gliomas expressed IL-17RA, with remarkable co-expression of the glioma stem cell (GSC) markers CD133, Nestin, and Sox2. IL-17 enhanced the self-renewal of GSCs as determined by proliferation and Matrigel^®^ colony assays. IL-17 also induced cytokine/chemokine (IL-6, IL-8, interferon-γ-inducible protein [IP-10], and monocyte chemoattractant protein-1 [MCP-1]) secretion in GSCs, which were differentially blocked by antibodies against IL-17R and IL-6R. Western blot analysis showed that IL-17 modulated the activity of signal transducer and activator of transcription 3 (STAT3), nuclear factor κ-light-chain-enhancer of activated B cells (NF-κB), glycogen synthase kinase-3β (GSK-3β) and β-catenin in GSCs. While IL-17R-mediated secretion of IL-6 and IL-8 were significantly blocked by inhibitors of NF-κB and STAT3; NF-κB inhibitor was more potent than STAT3 inhibitor in blocking IL-17-induced MCP-1 secretion. Overall, our results suggest that IL-17–IL-17R interaction in GSCs induces an autocrine/paracrine cytokine feedback loop, which may provide an important signaling component for maintenance/self-renewal of GSCs via constitutive activation of both NF-κB and STAT3. The results also strongly implicate IL-17R as an important functional biomarker for therapeutic targeting of GSCs.

## INTRODUCTION

Malignant glioma is the most common type of primary brain tumor with approximately 23,000 newly diagnosed cases per year in the USA [1–3]. In spite of aggressive surgery, radiotherapy and chemotherapy, malignant glioma remains one of the most lethal solid tumors with a median survival of only 15 months, leading to 13000 deaths per year in the USA [4]. Therefore, more studies are needed to understand the mechanisms of glioma progression, especially focusing on tumor-stromal interactions that favor tumor progression, in order to identify new molecular targets for designing more effective therapy for this deadly disease.

Increasing reports in the literature have led to a consensus that cancer stem cells (CSCs) represent novel and translationally relevant targets for cancer therapy [5, 6]. First characterized in 2003, glioma stem cells (GSCs) make up a small proportion (1–10%) of the cells in gliomas, but they drive tumor growth via various mechanisms [7–10], impart persistence with enhanced DNA damage repair programs [8, 11] that resist aggressive chemo- and radiation therapies, and also induce tumor recurrence [12–16]. Several reports have suggested that attenuation of GSC self-renewal or survival could indeed be a promising therapeutic strategy for glioblastoma [9, 10, 17–21]. Elucidating novel mechanisms of GSC self-renewal or expansion could ultimately lead to new strategies for the treatment of glioblastoma. Research on GSC maintenance and self-renewal thus far have mostly focused on cell intrinsic factors such as transcription molecules and microRNAs [8, 21–24] or cell-extrinsic growth factors like transforming growth factor β (TGF-β) and neurotrophins [25, 26]. Potential significance of inflammatory cytokines on glioma progression via their direct impact on GSCs remains unexplored.

Several studies on extra-cranial tumors have pointed to a critical role of inflammation in cancer initiation, progression, and recurrence [27–29]. Interleukin 17 (IL-17) is one of the most potent inflammatory cytokines and has been strongly implicated in inflammatory autoimmune disorders such as rheumatoid arthritis and multiple sclerosis [30, 31]. Our group and others have recently reported the prevalence of IL-17 secreting cells in malignant gliomas and studied the mechanism of their recruitment and immune functions in the tumor milieu [32–36]. We have observed, for the first time, preferential co-localization of IL-17RA (referred to as IL-17R in this manuscript) with GSC markers in human primary malignant gliomas, suggesting that IL-17R^+^ glioma cells may represent a subpopulation of GSCs. The objective for this study was to explore a novel inflammatory axis involving IL-17-IL-17R interaction as a potential mechanism for maintenance, self-renewal or stimulation of GSCs. A mechanistic insight into the role of IL-17 in glioma progression would provide important avenues for studies on therapeutic manipulation of this novel inflammatory axis in malignant tumors and may improve outcome.

## RESULTS

### Gliomas express IL-17R which preferentially co-localizes with GSC markers

**Figure 1:**
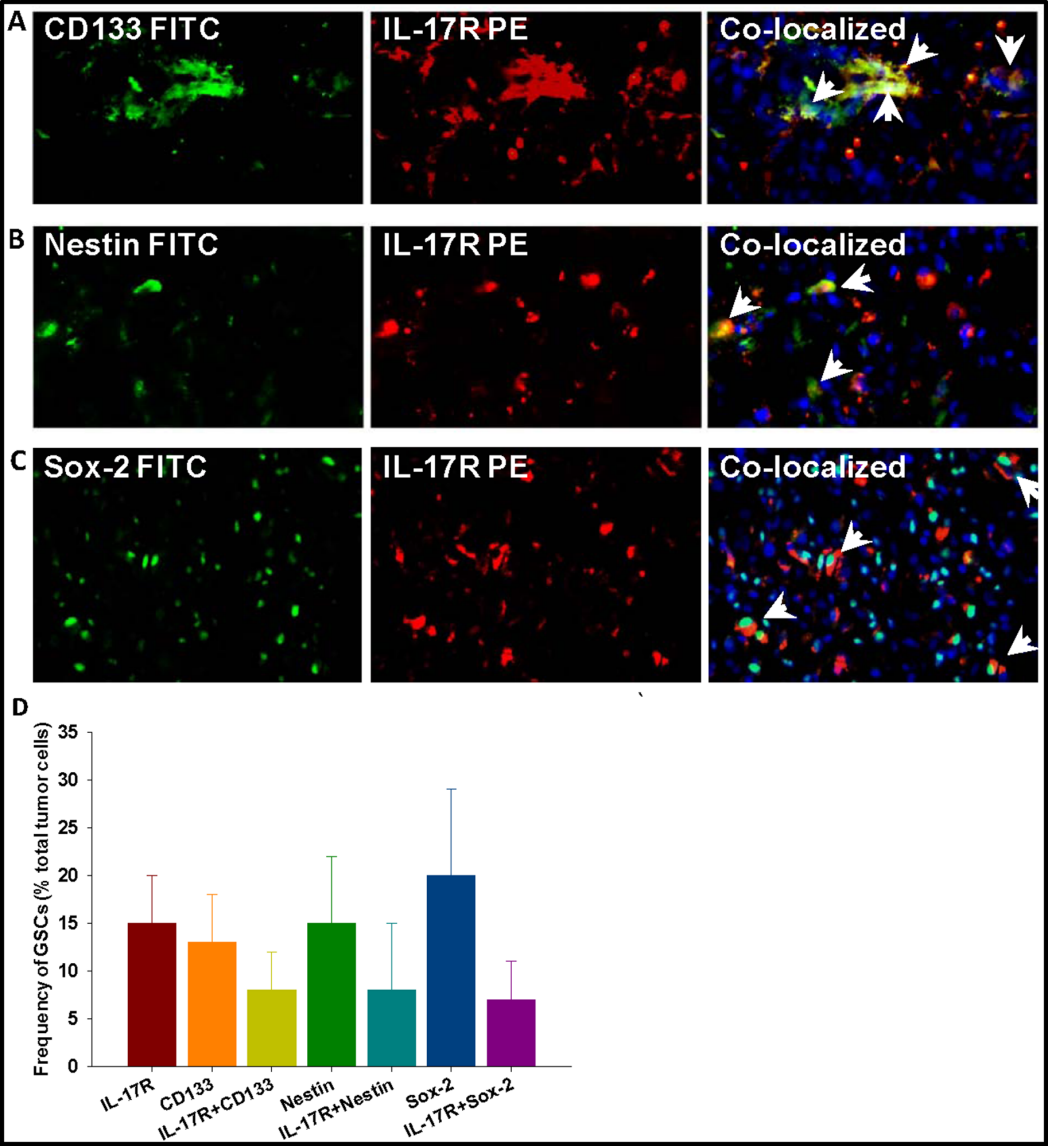
Gliomas express IL-17R which preferentially co-localizes with GSC markers Frozen tumor sections, obtained from patients with malignant glioma were analyzed by immunohistochemistry to determine the presence of IL-17R (**A**) in GSCs expressing CD133 (A, *top panel*), Nestin (A, *middle panel*), and Sox2 (A, *lower panel*). Result shown is from one representative frozen glioma specimen out of 12 studied. The micrographs were imaged at 200 × magnification. More than 100 nucleated (DAPI positive) cells were counted at three different fields per tissue section and at least two tissue sections were stained per every specimen. The histogram (**B**) shows cumulative frequency of cells (% total tumor cells) staining for IL-17R and the three GSC markers.

High grade glioma tissues were obtained from 12 patients (7 male and 5 female, aged between 45 and 73) with grade IV glioblastoma multiforme (GBM). As determined by immunohistochemical analysis, the tumor samples showed various range of reactivity to GFAP, EGFR and the proliferation marker ki67 (Supplementary Table 1). On average, 15% (range 2–19%) of malignant glioma cells expressed IL-17R and more than 60% of IL-17R^+^ cells co-expressed the GSC markers CD133, Nestin, and Sox2 (Figure 1 and Supplementary Figure S1). Similar to the transcription factor NF-κB, Sox2 has been shown to localize in the cytoplasm and translocate into the nucleus to regulate its transcriptional activity [37]. In the current study, the majority of Sox2 expression was observed in the nucleus while a few glioma cells had Sox2 localized in the cytoplasm. IL-17R was noted to co-localize with both cytoplasmic as well as nuclear expression of the Sox2 transcription factor (Figures 1 and S1).

**Figure 2:**
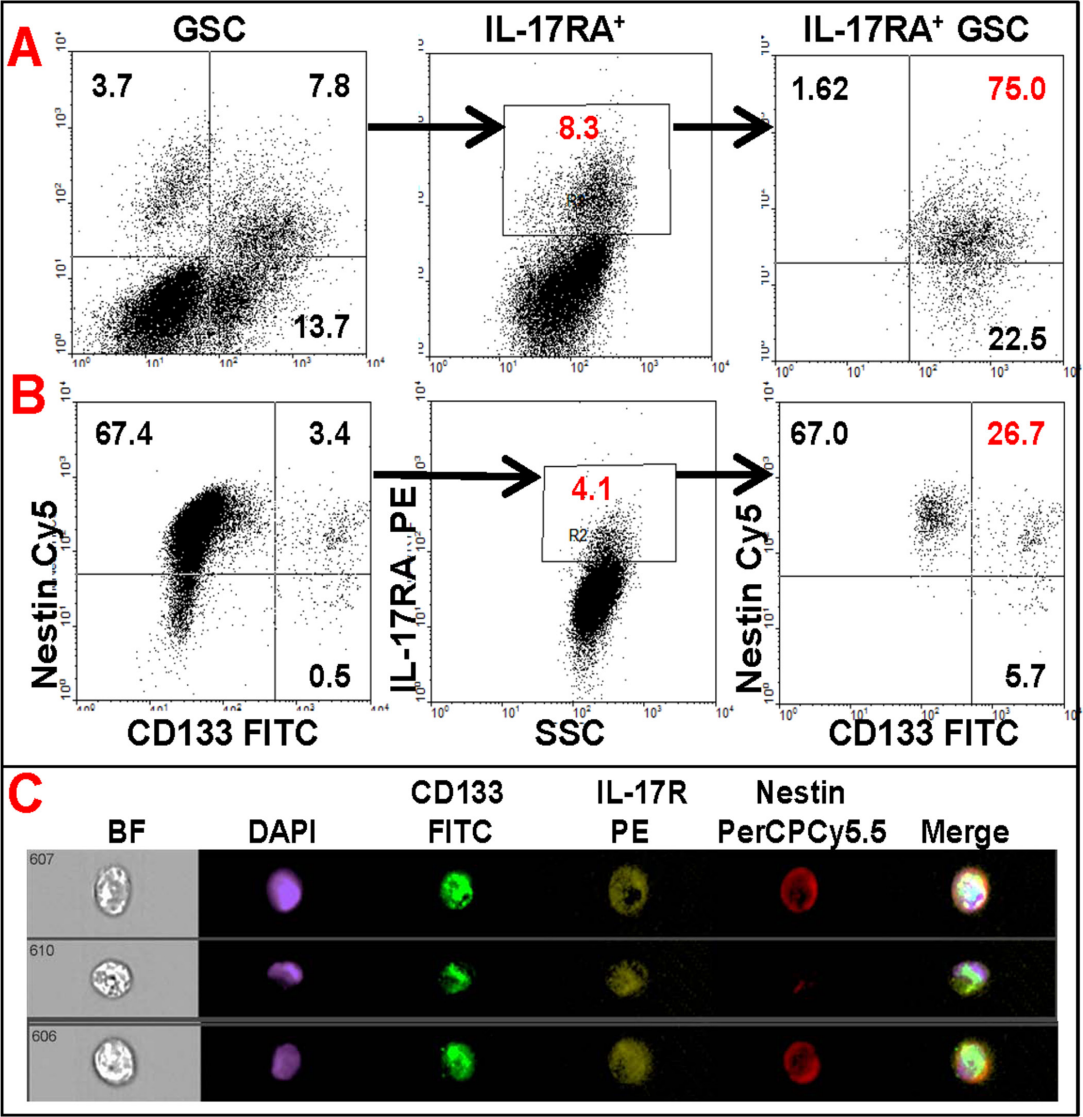
Flow cytometric analysis of primary glioma cells showing co-expression of IL-17R with GSC markers Freshly-dissociated or frozen total tumor cells obtained from patients with malignant glioma were analyzed by multi-color flow cytometry before culture (**A** and **C**) or after 7 passages through culture (**B**). The data are from one representative patient sample out of 5 studied.

In a parallel study, single cell suspensions, generated from 5 malignant glioma specimens were analyzed by multi-color flow cytometry before culturing, and therefore accurately representing the tumors’ cellular heterogeneity (Figure 2A and 2C), or after 7 passages in culture (Figure 2B). In agreement with the results of immunohistochemical analysis (Figure 1), flow cytometry analysis also revealed that an average of 12% (range 2–19%) of fresh (not cultured) tumor cells expressed IL-17R and 75% of IL-17R^+^ cells co-expressed GSC markers CD133 and Nestin (Figure 2A). On the other hand, although the relative population of IL-17R-expressing cells declined to about 4% (range 0.5–6%) after several passages through culture in serum-containing media, more than 25% of the IL-17R^+^ cells maintained co-expression of both Nestin and CD133 (Figure 2B). Flow cytometric analysis also confirmed co-expression of IL-17R and the transcription factor Sox-2, along with CD133 in patient-derived glioma cells (Supplementary Figure S2). These results strongly suggest that IL-17R could be a critical phenotypic marker for GSCs.

### IL-17 enhances the self-renewal of GSCs

**Figure 3:**
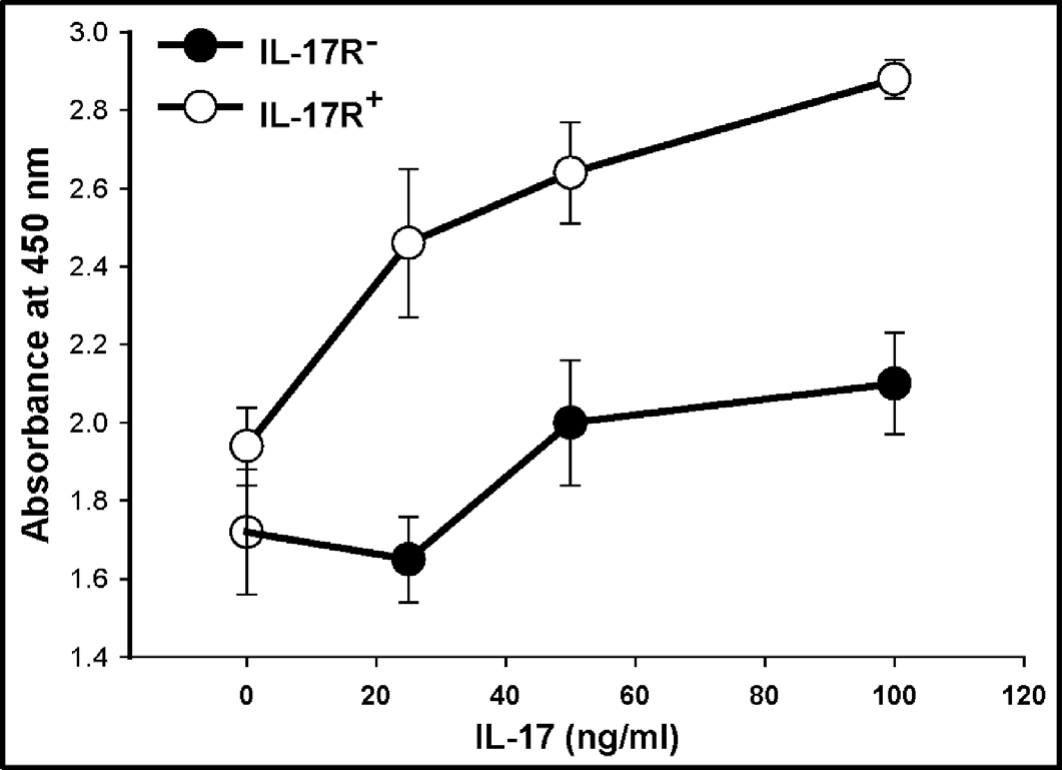
IL-17 enhances the proliferation of GSC-enriched IL-17R^+^ cells Primary glioma cells generated from malignant glioma specimens or U87-MG glioma cell line were seeded in 96-well plates (2 × 10^4^ cells/well) and cultured in the presence of IL-17. After 4 days, cell viability was evaluated by WST-1 assay. Cell viability was expressed as percent of control (cells cultured with medium alone). The data represents mean ± S.D. of two independent experiments, performed in triplicates, with similar results.

In order to explore the functions of IL-17R in malignant gliomas, we first assessed the effect of IL-17 on the proliferation of glioma cells. Primary glioma cells and U87-MG cells were cultured in the neurosphere medium in the presence of IL-17 (25 and 100 ng/ml) for 72 h. As determined by the WST cell proliferation assay, treatment with IL-17 did not significantly alter the proliferation of the primary glioma or the U87-MG cells (Supplementary Figures S3A and S3F, respectively), compared to the medium controls. However, when GSC-enriched, FACS-isolated IL-17R^+^ and IL17R^−^ cells were used for the experiment, IL-17 significantly and dose-dependently enhanced the proliferation of IL-17R^+^ GSCs, whereas the IL-17R^−^ culture group did not significantly respond to IL-17 (Figure 3).

We further examined whether IL-17 could affect the proliferation or self-renewal of the GSCs via a CFSE dissemination assay, as described in the Methods. Only the IL-17R^+^ GSCs (gated for CD133^+^IL-17R^+^) were analyzed for CFSE intensity. In Supplementary Figures S3C, S3E, S3H and S3J, the dividing cells are designated by cell division numbers 1, 2, 3, 4, and 5, where number 1 represents parent cell population (blue histogram). In primary glioma culture, there were 38% dividing and 61.7% parent GSCs in IL-17 treatment group (Figure S3E) compared to 34% dividing GSCs and 64% parent in medium control (Figure S3C). Similarly, in U87-MG culture, there were 85% dividing GSCs and 12% parent in IL-17 group (Figure S3I) compared to only 78% dividing GSCs and 20.2% parent in the control (Figure S3H). Thus, IL-17 treatment had enhanced the frequency of dividing cells among the GSC (CD133^+^IL-17R^+^) population in both primary glioma and U87-MG cultures, suggesting that IL-17 potentially enhances proliferation or self-renewal of GSCs.

**Figure 4:**
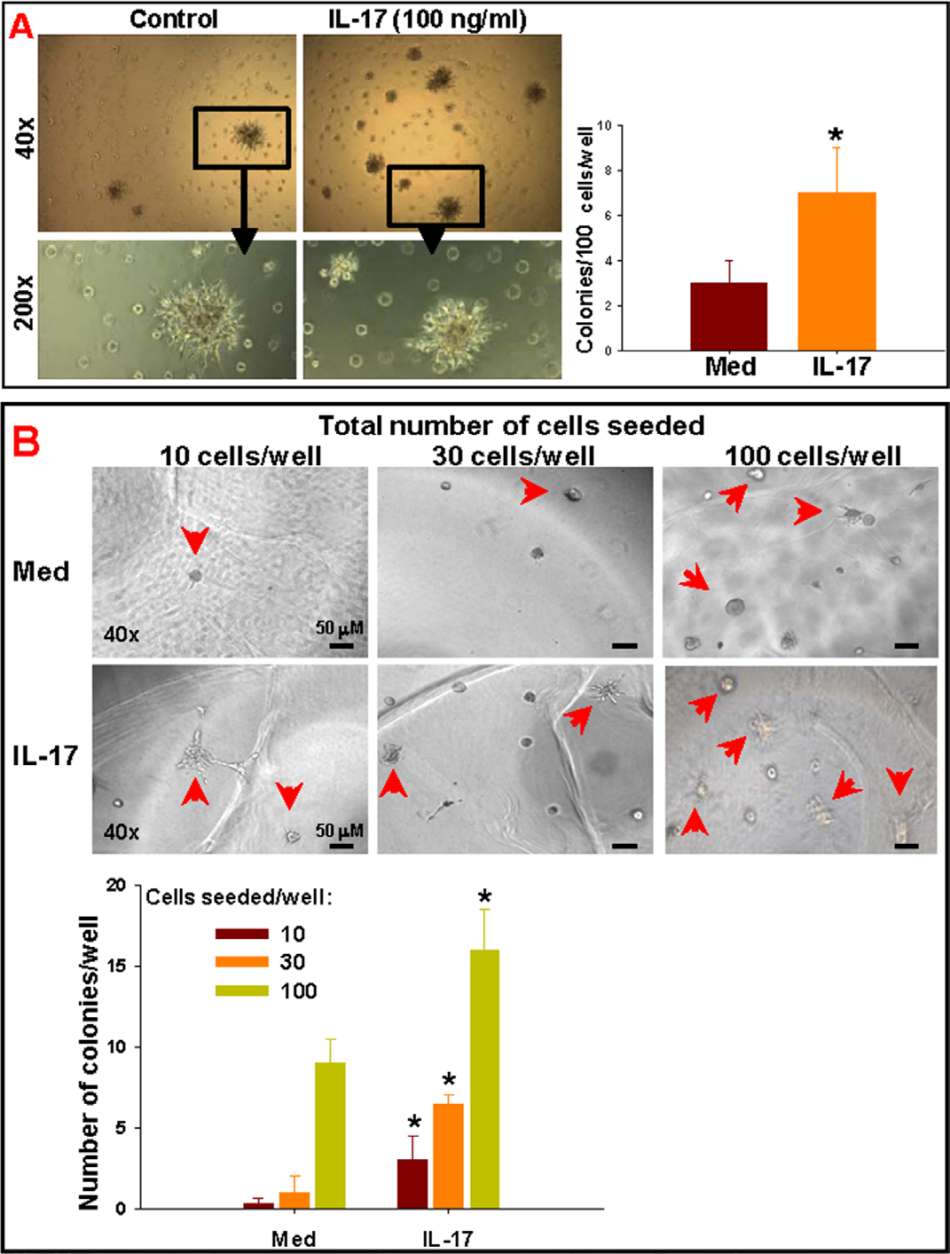
IL-17 enhances the self-renewal of GSCs Primary glioma cells, obtained from patients with malignant glioma, were enriched for GSC population via neurosphere culture followed by FACS-isolation of IL17R^+^ and IL-17R^−^ cells, as described in the Methods. (**A**) Total or (**B**) IL-17R^+^ GSC-enriched cells were seeded in Matrigel^®^ layers, in triplicates, in a 48-well plate (10, 30 or 100 cells/well, as indicated) and cultured in neurosphere media in the presence of IL-17. The GSC colonies (> 10 cells) were counted after 10 days. The data represents mean ± S.D. of three independent experiments with similar results. *indicates *p* < 0.05 *versus* Medium control.

To demonstrate that IL-17 indeed enhances the self-renewal of GSCs, a Matrigel^®^ colony forming assay was performed, as described in the Methods. In the first experiment, total GSC-enriched cells were seeded in Matrigel^®^ layers in triplicates in a 48-well plate (100 cells/well) and cultured in neurosphere media in the presence of IL-17. The GSC colonies (> 10 cells) were counted after 10 days. The cultures in medium control developed 3 ± 1 colonies/well; which was significantly (*p* < 0.05) enhanced to 7 ± 2 colonies/well upon treatment with IL-17 (Figure 4A). Next, we performed a limiting dilution colony assay with FACS-isolated IL-17R^+^ GSC-enriched cells. For IL-17R^+^ cells, the limiting dilution of cells to be plated for any colony to be observed in absence of exogenous IL-17 was 10 cells/well (Figure 4B), whereas the limiting dilution was 30 cells/well for IL-17R^−^ GSCs (data not shown). Moreover, when 100 cells were seeded, the number of IL-17R^−^ GSC colonies observed were 3 ± 1/well (Figure 4A), whereas almost 3 times more colonies (8 ± 3/well) were observed in IL-17R^+^ GSC groups (Figure 4B) even in absence of exogenous IL-17. Addition of IL-17 (100 ng/ml) significantly enhanced the number of GSC colonies in all dilutions tested (Figure 4B).

### IL-17 enhances the expression of stemness/mesenchymal markers in GSCs

**Figure 5:**
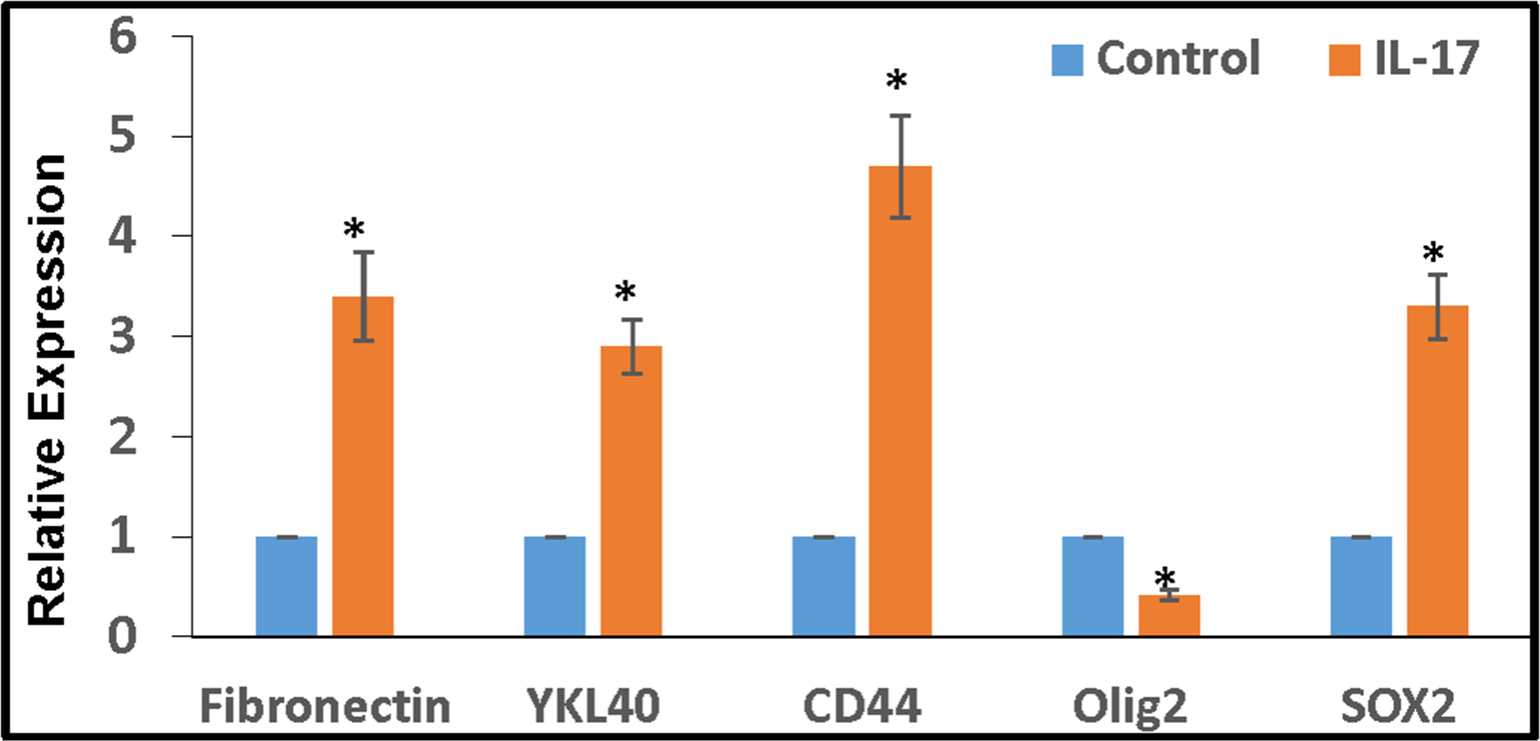
IL-17 enhances the expression of stemness/mesenchymal markers in GSCs Primary glioma cells, obtained from patients with malignant glioma, were cultured in stem cell medium for 2 weeks to obtain GSC neurospheres, as described. The GSCs were further cultured with IL-17 (100 ng/ml) for 3 days and the expression of stemness/mesenchymal markers were determined by RT-PCR.

GSCs were cultured in stem cell medium with IL-17 (100 ng/ml) for 3 days. As determined by quantitative RT-PCR, the expression of all stemness and mesenchymal markers tested, except for Olig2, were significantly enhanced by IL-17 (Figure 5).

These results clearly indicate that IL-17R in gliomas is functional and that IL-17–IL-17R interaction stimulates the self-renewal of GSCs. IL-17 also appears to alter glioma plasticity or induce stemness.

### IL-17–IL-17R interaction in GSCs mediates positive feedback loop of inflammatory cytokines involving multiple signaling pathways

**Figure 6:**
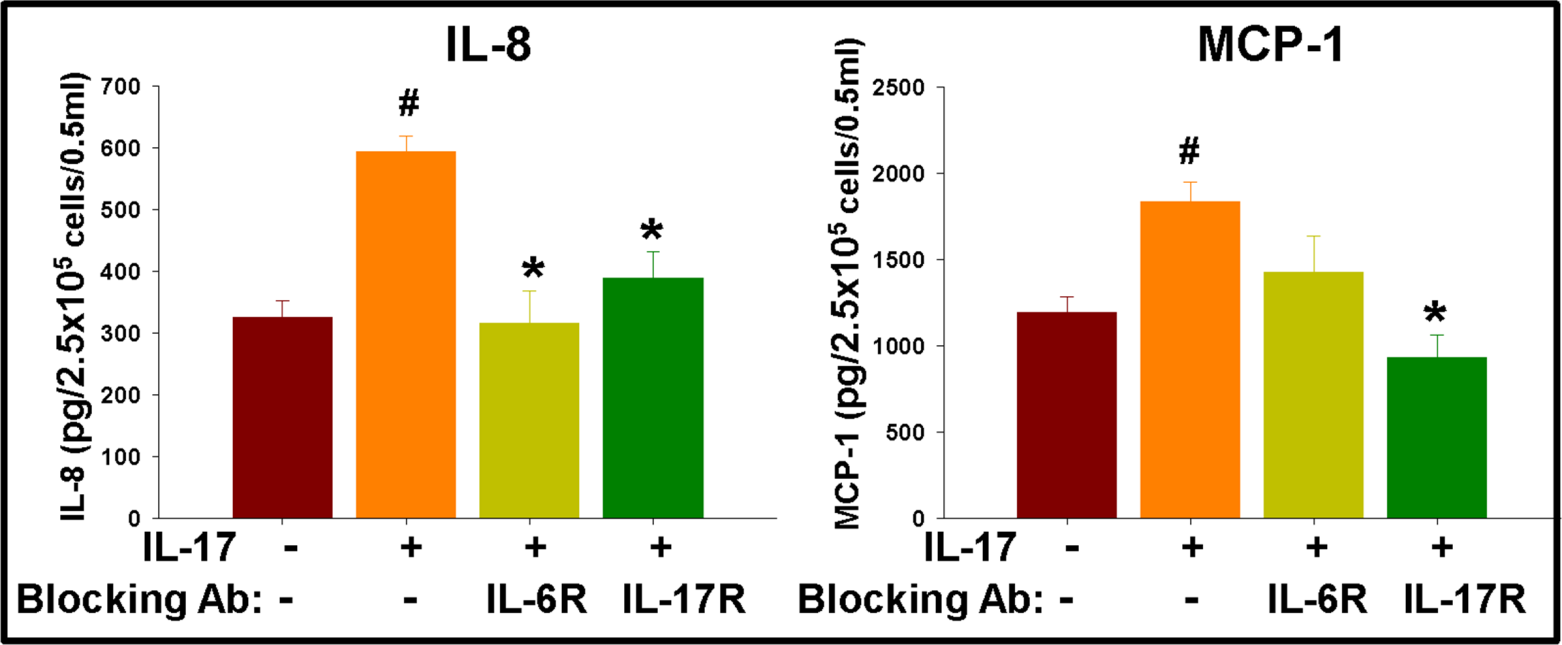
IL-17 enhances secretion of inflammatory cytokines in GSCs, which is regulated by cytokine feedback loop IL-17R^+^ GSCs were isolated from primary glioma cells via flow sorting and seeded at 2.5 × 10^5^ cells/0.5 ml/well into a 12-well plate in neurosphere medium, and then cultured with IL-17 (100 ng/ml), in the presence of blocking antibodies to IL-17R and IL-6R. After 72 h, the cytokines were measured in the culture supernatants using a cytokine Bio-Plex array. The data, expressed as pg/2.5 × 10^5^ cells/0.5 ml, are from one representative experiment out of three experiments performed with similar results. ^#^indicates *p* < 0.05 *versus* Medium control. *indicates *p* < 0.05 *versus* IL-17 alone group.

**Figure 7:**
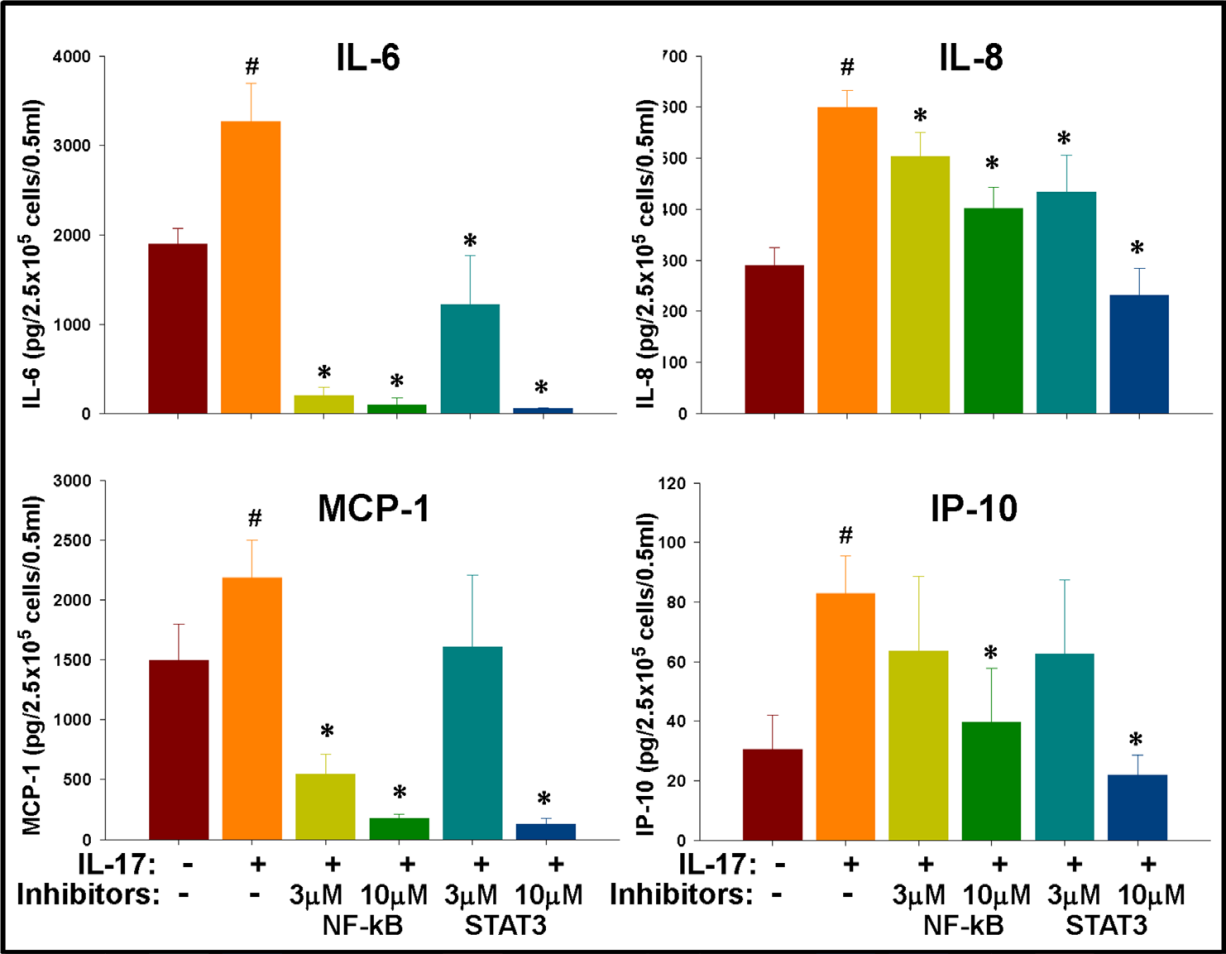
IL-17 mediated enhancement of inflammatory cytokines in GSCs is regulated by NF-κB and STAT-3 signaling IL-17R^+^ GSCs were isolated from primary gliomas via flow sorting and seeded at 2.5 × 10^5^ cells/0.5 ml/well into a 12-well plate in neurosphere medium and then cultured with IL-17 (100 ng/ml) in the presence of specific signaling inhibitors. After 72 h, the cytokines were measured in the culture supernatants using a cytokine Bio-Plex array. The data, expressed as pg/2.5 × 10^5^ cells/0.5 ml, are from one representative experiment out of three experiments performed with similar results. ^#^indicates *p* < 0.05 *versus* Medium control. *indicates *p* < 0.05 *versus* IL-17 alone group.

In order to further assess the functionality of IL-17R in GSCs and to elucidate the underlying mechanisms, IL-17R^+^ GSCs were isolated from primary gliomas via flow sorting and seeded at 0.25 × 10^6^ cells/ml/well into a 12-well plate in neurosphere medium, and then cultured with IL-17 (100 ng/ml) in the presence of IL-17R/IL-6R blocking antibodies (1 μg/ml) and specific signaling inhibitors, as indicated. After 72 h, the cytokines were measured in the culture supernatants using a cytokine Bio-Plex array, as described in the Methods. IL-17 significantly enhanced the secretion of IL-6, IL-8, IP-10, and MCP-1 by the GSCs (Figures 6 and 7). While IL-17-induced secretion of IL-8 was significantly (*p* < 0.05) blocked by antibodies to both IL-17R and IL-6R, MCP-1 secretion was significantly inhibited only by blocking IL-17R and not IL-6R (Figure 6). We also observed a basal level of IL-17 secretion (50–100 pg/0.25 × 10^6^ cells/0.5 ml) by GSCs, which was undetectable in anti-IL-6R treated groups (data not shown).

In the next experiment, both STAT3 inhibitor VI (Calbiochem, San Diego, CA) and InSolution^™^ NF-κB activation inhibitor (Calbiochem) significantly (*p* < 0.05) reversed the IL-17-induced secretion of IL-6 as well as IL-8 at both doses tested (Figure 7). NF-κB inhibitor was more potent than the STAT3 inhibitor in reversing IL-17-induced secretion of MCP-1; while both NF-κB and STAT3 inhibitors reversed the secretion of IP-10 only at the higher dose tested (Figure 7).

In agreement with the proliferation/colony formation data, these results clearly indicate that the IL-17R in the GSCs are functional and that IL-17R induced cytokine secretion may be differentially regulated by cytokine feedback loops involving NF-κB and STAT3 signaling.

**Figure 8:**
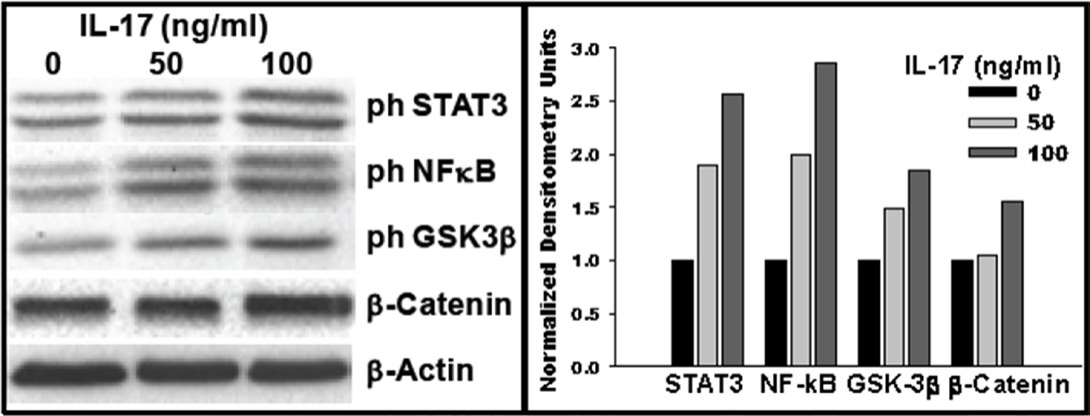
IL-17–IL-17R interaction in GSCs activates multiple signaling pathways GSCs were seeded into a 12-well plate in neurosphere medium in presence of IL-17, as indicated. After 72 h, cells were lysed and proteins analyzed by western blotting. Grayscale density was analyzed using ImageJ software and values for signaling molecules were normalized against the total protein content and those for β-actin. Data are representative of three experiments performed with similar results.

Next, GSCs were seeded into a 12-well plate in neurosphere medium in presence of IL-17. After 72 h, cells were lysed and proteins analyzed by western blot. GSCs showed moderate basal phosphorylation of STAT3 and NF-κB (p65), which was enhanced 2.5-fold and 2.8-fold, respectively, with IL-17 treatment at 100 ng/ml (Figure 8). Treatment with IL-17 also enhanced β-catenin expression by 1.5-fold, which positively associated with about 2-fold increase in phosphorylation (inactivation) of glycogen synthase kinase-3β (GSK-3β) (Figure 8).

The results indicate that stimulation of GSCs by IL-17 is potentially regulated by multiple signaling pathways.

## DISCUSSION

The cytokine profile as well as the type and density of immune cells in the tumor microenvironment have been shown to play a critical role in the progression of malignant tumors [38–40]. Our group and others have demonstrated the infiltration/enhancement of IL-17-producing cells in malignant gliomas [32–34, 36] and IL-17 has been linked to cancer progression in many tumor types [33, 41–44]. IL-17 over-expression potentially enhance glioma cell growth *in vivo*, which may be associated with accelerated angiogenesis [45]. The sera of patients with malignant glioma have been reported to have significantly elevated levels of IL-17A, compared to those harboring a meningioma or schwannoma [46]. Studies using tumor tissues from glioma patients have, however, shown conflicting results. A recent study on immunohistochemical analysis of tumors from 41 patients with glioblastoma noted that patients with ‘high levels’ of IL-17 expression had significantly longer overall survival than those with ‘low levels’ of tumoral IL-17 expression [47]. However, the study design and interpretation of data were somewhat controversial, because the patients were followed for only 24 months, and the criteria for ‘low IL-17 expression’ (less than 15% of the total tumor cells) was quite unrealistic. On the other hand, a flow cytometric analysis of glioma tissues from 31 patients showed the presence of immune-suppressive IL-17^+^FoxP3^+^ T cells (8% in gliomas versus 0.5–1% in control tissues) [48]. More importantly, both these studies did not analyze the status of IL-17R expression in the tumor cells. Overall, the role of IL-17 in glioma progression remains uncertain and the precise underlying mechanisms remain largely unknown [33]. In the current study, we have demonstrated a potential impact of IL-17-IL-17R interaction in glioma growth and progression via direct stimulation of the GSCs.

Our results from the analysis of tumor tissues from patients with malignant gliomas have demonstrated preferential expression of IL-17R in glioma cells which also expressed GSC markers CD133, Nestin, and Sox2. GSCs were first identified and purified from human brain tumors by Singh et al. as a population of CD133^+^ tumor cells [49, 50]. While several studies have reaffirmed strong association of CD133 with GSCs, the intermediate filament protein Nestin and the transcription factor Sox2 have also often been reported in GSCs [8, 33, 51–54]. Sox2 plays a critical role in the induction or maintenance of glioma ‘stemness’ [8, 55]. Expression of Sox2 and Ki-67 were also negatively correlated with overall survival and progression-free survival [56]. In the current study, since we observed the majority (60% or more) of the IL-17R^+^ glioma cells were also positive for one or more of these GSC markers, it is quite plausible that the IL-17R^+^ glioma cells constitute a population of GSCs and IL-17 may regulate glioma progression via direct influence on GSCs.

Our results demonstrate that IL-17R in GSCs is indeed functional and can play an important role in the maintenance of GSCs by inducing their proliferation or self-renewal. The induction of proliferation by IL-17 was not apparent in fresh gliomas or in cell lines before they were enriched for GSCs. However, significant induction of proliferation by IL-17 was observed in GSC-enriched and FACs-isolated IL-17R^+^ cells. The CFSE dissemination analysis also revealed increased cell division among a small population of IL-17R^+^CD133^+^ GSCs following treatment with IL-17. Moreover, IL-17R^+^ GSC-enriched cells showed significantly higher capacity for colony formation in Matrigel^®^, compared to IL-17R deficient cells and the number of colonies were significantly enhanced in the presence of IL-17. The results corroborated with the proliferation data and confirmed that IL-17 could stimulate or enhance the self-renewal of GSCs. RNA analysis of GSCs also revealed increased expression of mesenchymal/stemness markers (Fibronectin, YLK-4, CD44 and Sox2) following treatment with IL-17, suggesting that IL-17 may play a role in altering the plasticity of gliomas by inducing ‘stemness’ or enhancing glial-mesenchymal transition. Further studies are needed to confirm the same. It is to be noted that in all these experiments, cells were cultured in neurosphere media containing EGF and bFGF. In other cell types, IL-17 has been shown to induce epidermal growth factor receptor (EGFR) expression and also regulate EGFR signaling via receptor transactivation [57–59]. Further studies are warranted to examine whether any of these mechanisms are associated with IL-17R mediated stimulation of GSCs.

Our results are also in agreement with recent reports on extra-cranial tumors where stimulation of cancer stem cell by IL-17 has been observed. In an interesting study by Lotti et al, chemotherapy has been shown to induce IL-17 secretion in colorectal cancer associated fibroblasts, and IL-17 in turn, stimulated self-renewal of cancer stem cells while also promoting *in vivo* tumor growth [60]. Concurrent with our study, Xiang et al. have also observed stimulation of CD133^+^ ovarian cancer stem cells by IL-17 and enhanced tumor formation in nude mice models [61]. These studies along with the one presented herein clearly suggest a critical role for IL-17-IL-17 interaction in select tumor types, including malignant gliomas. While this may provide an opportunity for further studies into targeting IL-17R itself for therapeutic attenuation of some GSC population, more studies into the mechanism of IL-17−IL-17R interaction may reveal other molecular targets downstream of IL-17R pathway.

We observed enhanced secretion of IL-6, IL-8, IP-10, and MCP-1 by GSCs in the presence of IL-17. Generally, these cytokines are known to enhance tumor infiltration or expansion of tumor-promoting immune cells while also inducing angiogenesis and invasion [62, 63]. Fischbach et al. have recently demonstrated a role for IL-8-mediated interaction between endothelial cells and perivascular GSCs in regulating the migration, growth, and maintenance of stemness [64]. IL-6 is one of the most studied cytokines in gliomas and has been associated with tumor growth, angiogenesis, and poor survival [65–67]. Since IL-6 is also known to enhance secretion of several chemokines/cytokines in tumor cells, we examined whether some of the effects of IL-17 were mediated indirectly via enhancement of IL-6 secretion. Interestingly, IL-17-mediated enhancement of IL-8 secretion was significantly reversed by antibodies to IL-17R and IL-6R as well. On the other hand, while the secretion of MCP-1 was significantly reversed by blocking IL-17R, inhibition by IL-6R antibody was only modest, but not significant. The results suggest that while IL-17 could influence cytokine secretion via regulation of IL-6 activity, it did also have distinct, IL-6-independent effect on cytokine secretion by gliomas.

Our results further showed significant blocking of IL-17-induced IL-6 and IL-8 secretion by specific NF-κB inhibitor. These findings are consistent with an earlier study that showed involvement of IL-17–NF-κB signaling in enhanced secretion of IL-6 and IL-8 in glioma cell lines [68]. In addition, secretion of IL-8 was also significantly reversed by STAT3 inhibitor at both doses tested. Overall, our results clearly indicate that at least IL-8 induction by IL-17 in gliomas is mediated via IL-6/STAT3 axis. Our results are in agreement with a recent report by McFarland et al. [69], which showed that STAT3 activation and STAT3 target gene expression by tumor necrosis factor α (TNF-α) is affected through NF-κB induced expression of IL-6 in glioblastoma cell lines as well as in an *in vivo* xenograft model. In our study, IL-17 dependent secretion of MCP-1 and IP-10 were inhibited by both NF-κB and STAT3 inhibitors only at higher doses (10 μg/ml), suggesting that the induction may be regulated by an STAT3/NF-κB independent signaling pathway.

Our western blot analyses did reveal enhanced phosphorylation (inactivation) of GSK-3β and enhancement of β-catenin, along with NF-κB and STAT3 signaling in GSCs following treatment with IL-17. Inactivation of GSK-3β stimulates β-catenin, which in turn potentially regulates GSC differentiation and tumor progression via regulation of Notch pathway [70, 71]. The GSk-3β/β-catenin pathway may represent the NF-κB/STAT3 independent pathway downstream of IL-17R in GSCs.

MCP-1 has been associated with tumor infiltration of monocytes/microglia, potentially resulting in glioma aggressiveness and poor prognosis [72, 73]. The role of IP-10 (CXCL-10) has not been well studied in relation to progression of gliomas. However, a recent study by Rezaeifard et al. [74] has implicated adipose stem cell derived IP-10 in the pathogenesis of ovarian cancer. Further studies in this direction may provide valuable insight into alternative or additional mechanisms by which IL-17–IL-17R interaction could promote GSC stimulation and glioma progression.

Constitutive activation of both NF-κB and STAT3 is critical for maintenance of GSC functionality and maintenance of stem-like property [19, 24]. While STAT3 has been known to activate NF-κB via direct interaction with NF-κB/p65 [75], it is unclear whether a reverse scenario is possible via similar interaction in GSCs. The current study provides preliminary evidence that IL-17–IL-17R interaction may contribute to activation of both STAT3 and NF-κB stimulation via a positive cytokine feedback loop involving IL-17 and IL-6. Studies are under way in order to reveal the precise molecular mechanisms that regulate the functions of IL-17 in GSCs.

Lastly, there has been considerable interest in developing novel pharmacologic inhibitors of NF-κB and STAT3 for the treatment of malignant gliomas [69, 76, 77]. It is also being increasingly evident that inhibition of both NF-κB and STAT3 is necessary for an effective treatment of gliomas, at least in animal models [24, 69]. However, NF-κB and STAT3 are critical for functions of several normal cells, including immune cells. Therefore, identifying therapeutic targets upstream of these transcription factors, especially at the receptor level, is likely to yield better clinical efficacy against malignant gliomas.

## MATERIALS AND METHODS

### Glioma tissues, primary tumor culture, and cell lines

All human materials were used in accordance with the policies of the Institutional Review Board (IRB) at Wayne State University. Tumor tissues were obtained from patients with histologically-confirmed glioblastoma immediately after surgical resection, under a protocol approved by the IRB (#111610M1E). Depending on the amount of tumor tissue available, a part or whole of the tumor tissue was fixed with 4% paraformaldehyde, saturated with 30% sucrose and frozen in O.C.T. solution (Sakura Finetek USA, Torrance, CA) prior to sectioning for immunohistochemical analysis. The remaining portion was enzymatically digested to prepare single cell suspensions of primary glioma cells using the gentleMACS^®^ dissociator system with the Human Tumor Kit (Miltenyi Biotec, San Diego, CA) using the manufacturer's protocol. Some of the dissociated tumor cells were cultured in DMEM/F12 containing 10% FBS to derive primary glioma cell lines, while the bulk of patient-derived glioma cells were used in the studies described below. U87-MG cells were obtained from American Type Culture Collection (ATCC, Manassas, VA) and maintained in DMEM/F12 containing 10% FBS.

### Immunohistochemistry

A published protocol [78] was followed with some modifications. Briefly, the frozen tumor specimens were cut into 5 μm tissue sections, fixed in acetone for 5 min and stored at −80°C. For immunostaining, slides were brought to room temperature, blocked and hydrated in staining buffer (PBS with 5% goat serum) and appropriate primary antibodies (purified anti-CD133 and anti-IL-17RA from Santa Cruz Biotechnology, Santa Cruz, CA; anti-Nestin and anti-sex determining region Y-box 2 [Sox2] from eBioscience, San Diego, CA;) were applied, followed by overnight incubation at 4°C. For co-localization, appropriate secondary antibodies conjugated with FITC or PE (Santa Cruz Biotechnology) were utilized. Nuclei were stained with DAPI. Negative staining was performed with appropriate isotype control antibodies (eBioscience) instead of the specific primary antibody. Sections were then analyzed under a fluorescent microscope equipped with a digital camera (Olympus BX51) and micrographed at 200 × magnification. The numbers of cells with respective immunostaining were enumerated after counting 100 cells with distinct nuclear staining in at least three consecutive high-power fields in each slide, and in two tissues per slide. Necrotic or thick areas and severely overlapping tumor cells were excluded. The frequencies of cells expressing IL-17R alone or co-expressed with one of the GSC markers were calculated as percentage of the total number of nuclear-stained cells counted.

### Flow cytometry

Fluorescein-conjugated human antigen-specific or isotype control antibodies and anti-human IL-17RA PE were purchased from eBioscience; CD133 FITC and Nestin PerCPCy5.5 were from BD Bioscience, San Jose, CA; CD133 APC from Miltenyi Biotec; and Sox2 FITC was purchased from eBioscience. A published protocol was followed for surface and intracellular staining of the cells [79]. Tumor tissues obtained from 5 patients with histologically-verified glioblastoma were studied.

### GSC spheroid culture and isolation of IL-17R^+^ cells

A previously described protocol [54] was followed with some modifications. The glioma cells were cultured in neurosphere medium (DMEM-F12 1/1 containing glutamine 10 mM, HEPES buffer 10 mM and sodium bicarbonate 0.025% along with B-27 supplement (Life Technologies, Gaithersburg, MD), basic fibroblast growth factor (bFGF; 10 ng/ml) and epidermal growth factor (EGF; 20 ng/ml). After 14 days, the spheroids were dissociated using the FACSMax cell dissociation solution (Genlantis, San Diego, CA) as per the manufacturer's instructions. Dead cells were separated following a percol-gradient centrifugation. Single cell suspensions were analyzed for GSC phenotype, as described above, and either further cultured in neurosphere medium for the maintenance of GSC spheroids or used for experiments. For some studies, the GSC-enriched neurosphere derived cells were stained with IL-17RA PE (eBioscience) and the IL-17R^+^ and IL-17R^−^ cells were isolated using FACSaria II Cell Sorter (BD BioSciences).

### Cell proliferation WST assay

Cells were seeded to 96-well flat-bottom plates (2 × 10^4^ cells/well), and cultured in the presence of IL-17. After incubation at 37°C for 4 days, cell viability was evaluated using the WST-1 assay kit (Clonetech, Mountain View, CA) as per the manufacturer's protocol. The absorbance was measured at 450 nm, with the background correction at 600 nm, in an Infinite-200 microplate reader (Tecan Systems Inc., San Jose, CA). Cell viability/proliferation was expressed as a percent of control (cells cultured with medium alone).

### Cell proliferation CFSE dissemination assay

A published protocol [80] was followed with some modifications. Carboxyfluorescein diacetate succinimidyl ester (CFSE)-labeled primary glioma cells were plated (1 × 10^5^ cells/well) in a 48-well round bottom plate in the presence of IL-17. The cultures were incubated for 4 days at 37°C and then analyzed by flow cytometry. The number of cell divisions as a measure of cell proliferation was evaluated using the Proliferation Wizard Module in the ModFit LT Macintosh software (Verity Software House, Topsham, ME).

### GSC Matrigel^®^ colony forming assay

GSC-enriched cells were suspended in neurosphere medium with 0.2% Matrigel^®^ (BD Biosciences) and overlaid onto 0.5 mm thick bottom Matrigel^®^ in a 48-well plate at various number of cells/well. After 10 days of culture in the presence of IL-17, the GSC colonies (> 10 cells) were enumerated under a phase-contrast microscope.

### Real-time quantitative PCR analysis

RT-PCR was performed following a method described elsewhere [81]. Total RNA was isolated using TRIzol reagent (Invitrogen, Grand Island, NY) per the manufacturer's protocol and 1 μg of RNA was used to synthesize cDNA by SuperScriptase III (Invitrogen) with random primers. To detect the mRNAs of the different genes examined, the SYBR green method was used with the following primers:

Sox2, forward: TGGGTTCGGTGGTCAAGTC; reverse: CGCTCTGGTAGTGCTGGGA

OLIG2, forward: CTCCTCAAATCGCATCCAGA; reverse: AGAAAAAGGTCATCGGGCTC

YKL-40, forward: TGCCCTTGACCGCTTCCTCT; reverse: TTGATGAAAGTCCGGCGACT

CD44, forward: CTCCACCTGAAGAAGATTGT; reverse: AAGATGTAACCTCCTGAAGT

Fibronectin, forward: ACTGAGACTCCGAGTCA GCC reverse: TTCCAACGGCCTACAGAATT

S12, forward: TGCTGGAGGTGTAATGGACG; reverse: CAAGCACACAAAGATGGGCT

Average level of S12 RNA was used as an internal control.

### Cytokine analysis

Cytokines were measured in the culture supernatants using a 25-plex human cytokine Luminex Array (Invitrogen) and Bio-Plex system (Bio-Rad Lab, Hercules, CA). The multiplex panel includes IL-1β, IL-6, IL-8, IL-10, IL-17, IP-10, and MCP-1. The limit of detection for these assays was < 10 pg/mL based on detectable signal of > 2 fold above background (Bio-Rad). Cytokine concentration was automatically calculated from a standard curve by the BioPlex Manager Software (Bio-Rad).

### Western blot analysis

Western blot analysis of the protein samples were performed as described elsewhere [78]. Briefly, 20–30 μg aliquots of total protein were electrophoresed, transferred onto PVDF membrane, and probed with specific antibodies against phosphorylated and non-phosphorylated forms of signaling/transcription molecules (Cell Signaling Technology, Danvers, MA). Detection of HRP-conjugated antibodies was performed using SuperSignal (Pierce, Rockford, IL) and chemiluminescence recorded using an Omega 12iC Molecular Imaging System (UltraLum Inc., Claremont, CA).

### Statistical analysis

A Wilcoxon's log-rank test was performed to determine the statistical difference between various experimental and control groups using the SPSS package (SPSS Inc, Chicago, IL) [82]. A *p* value less than 0.05 was considered significant.

## CONCLUSIONS

**Figure 9:**
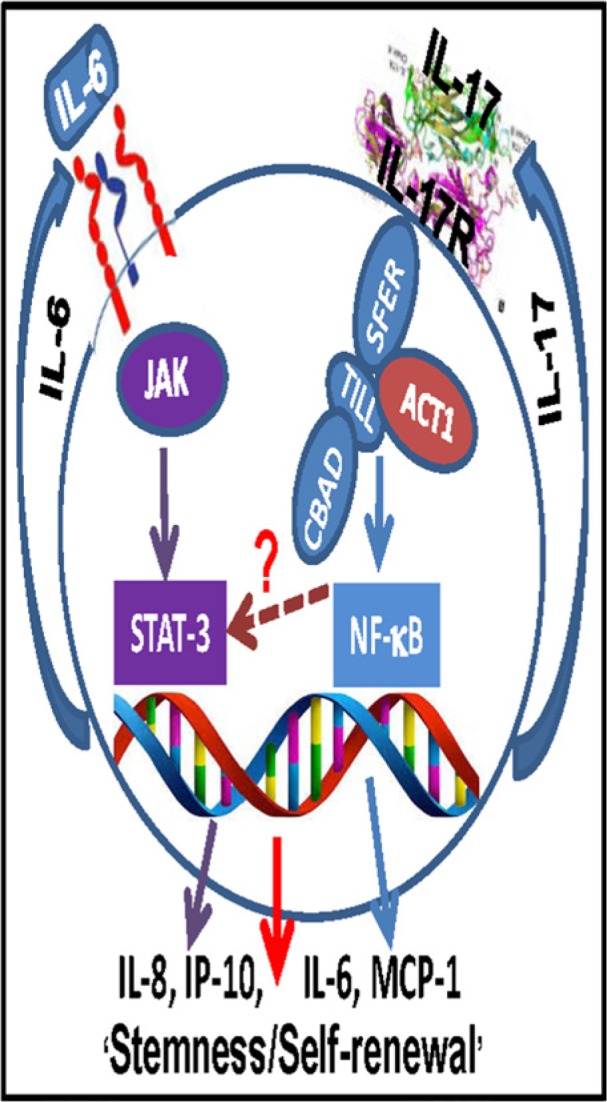
Schematic representation showing potential mechanism of IL-17R-mediated stimulation of GSCs IL-17-IL-17R interaction in GSCs potentially induces an autocrine/paracrine cytokine feedback loop, which in turn may provide a critical signaling component for maintenance/self-renewal of GSCs via constitutive activation of both NF-κB and STAT3.

We report here, for the first time, preferential expression of functional IL-17R in GSCs derived from primary human gliomas. Our results indicate that GSCs may be directly influenced by IL-17 during the initiation or progression of malignant gliomas. IL-17–IL-17R interaction in GSCs potentially induces an autocrine/paracrine cytokine feedback loop, which in turn may provide a critical signaling component for maintenance/self-renewal of GSCs via constitutive activation of both NF-κB and STAT3 (Figure 9). Further studies are needed to identify the precise molecular mechanisms downstream of IL-17R in GSCs. Identification of IL-17R as a functional molecular target on the surface of GSCs, as presented here, could be a crucial first step for larger studies towards developing unique therapeutic strategies aimed at either physical (cytotoxicity targeting IL-17R expression) or functional (blocking IL17–IL-17R axis) attenuation of the GSCs.

## SUPPLEMENTARY MATERIALS FIGURES AND TABLE


