# Supplementary material for: Preferential expression of functional IL-17R in glioma stem cells: potential role in self-renewal
Source: Oncotarget. 2016 Jan 8;7(5):6121–35. doi: 10.18632/oncotarget.6847 (PMC4868744; doi:10.18632/oncotarget.6847)
Supplement: Supplementary file 1 [file oncotarget-07-6121-s001.pdf]

Preferential expression of functional IL-17R in glioma stem cells: potential role in self-renewal

Supplementary Materials

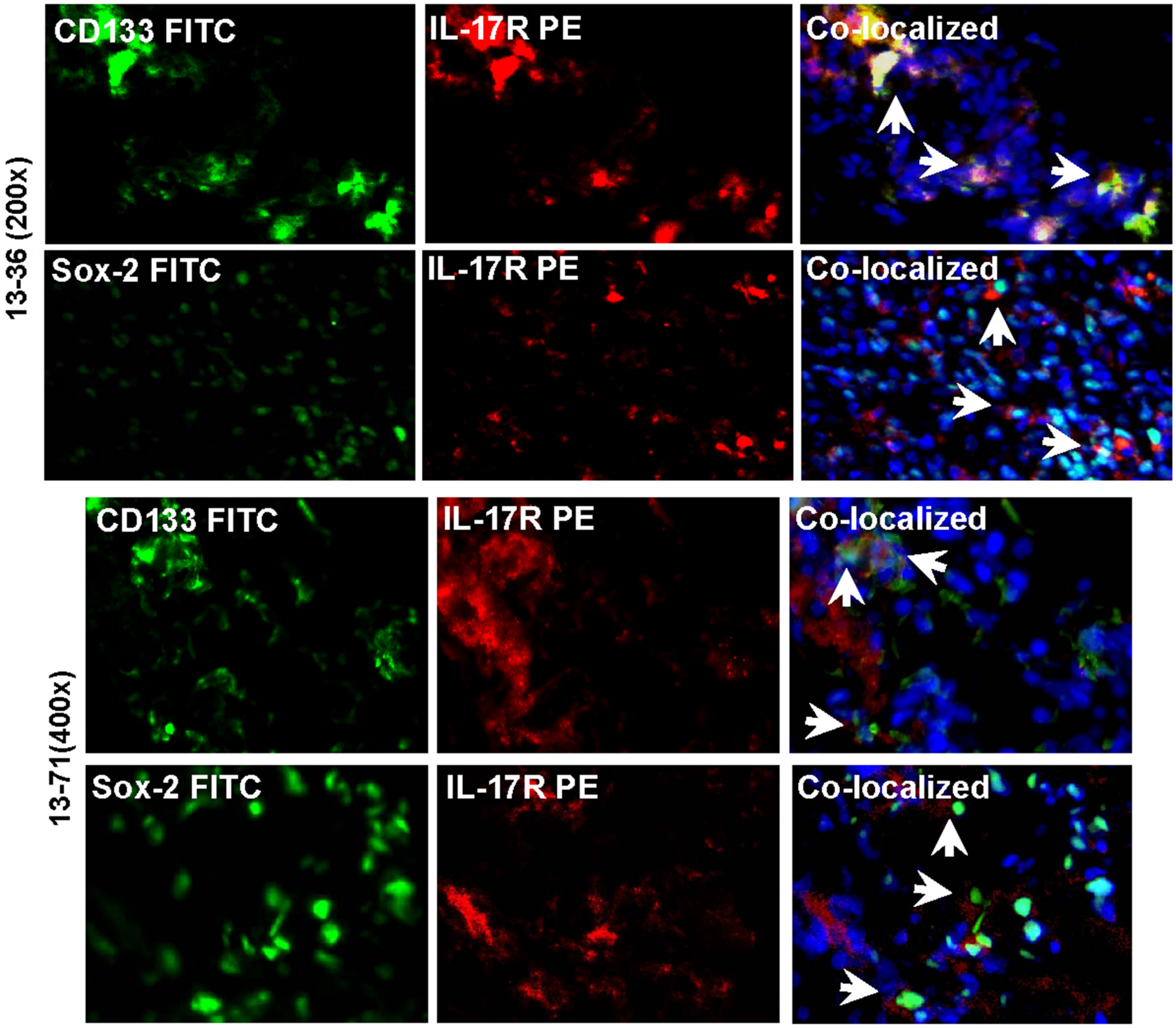

Supplementary Figure S1: Gliomas express IL-17R which preferentially co-localizes with GSC markers. Frozen tumor sections, obtained from patients with malignant glioma were analyzed by immunohistochemistry to determine the presence of IL-17R (**A**) in GSCs expressing CD133 (A, *top panel*) and Sox2 (A, *lower panel*). The data is from a glioma specimen derived from a different patient than the one shown in Figure 1.

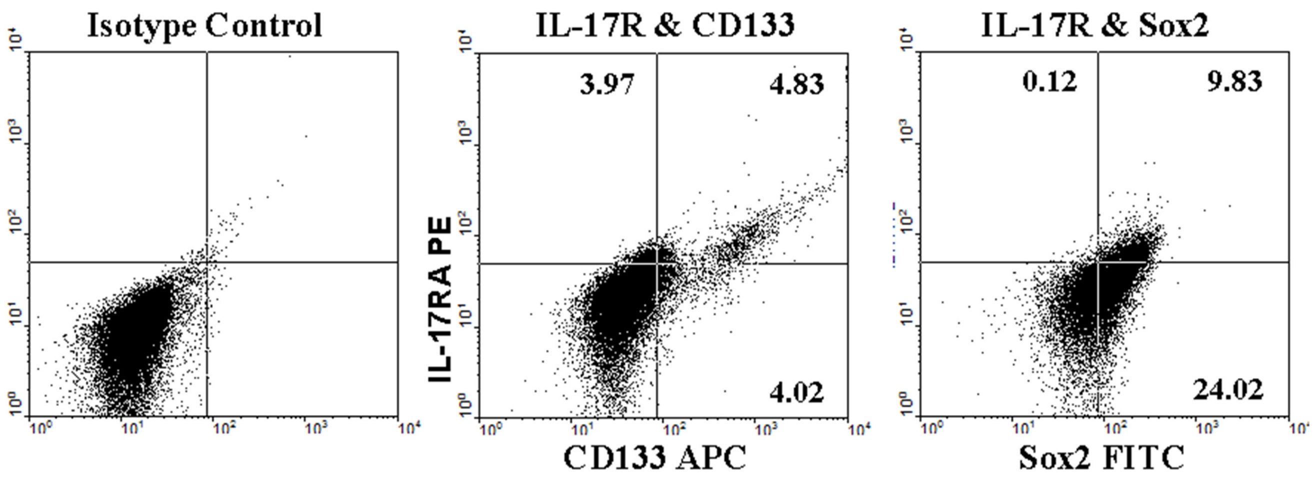

Supplementary Figure S2: Flow cytometric analysis of primary glioma cells showing co-expression of IL-17R with GSC markers. Freshly-dissociated or frozen tumor cells obtained from patients with malignant glioma were analyzed by multi-color flow cytometry before culture (**A** and **C**) or after 7 passages through culture (**B**). The data is from a glioma specimen derived from a different patient than the one shown in Figure 1.

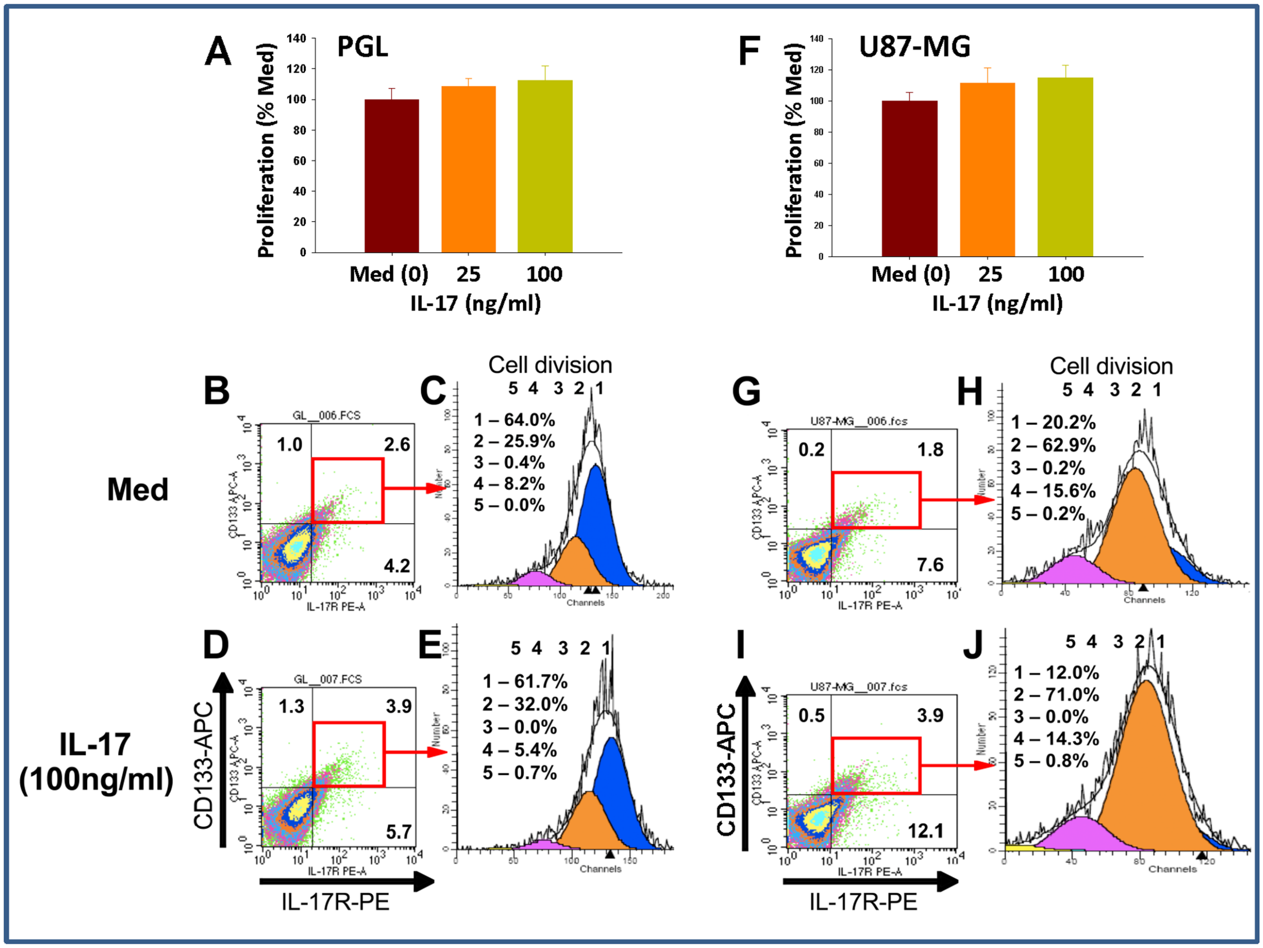

Supplementary Figure S3: IL-17 enhances the proliferation/self-renewal of GSCs. Primary glioma cells generated from malignant glioma specimens or U87-MG glioma cell line were seeded in 96-well plates (2×10^4 cells/well) and cultured in the presence of IL-17. After 4 days, cell viability was evaluated by WST-1 assay. Cell viability was expressed as percent of control (cells cultured with medium alone) and are shown in (**A** and **F**). The data represents mean ± S.D. of three independent experiments with similar results. Proliferation or self-renewal of GSCs was also evaluated by CFSE dissemination assay. CFSE-labeled primary glioma cells (**B**–**E**) and U87-MG cells (**G**–**J**) were plated in a 48-well round bottom plate in the presence of IL-17 (1×10^5 cells/well). The cultures were incubated for 4 days at 37°C and then analyzed by flow cytometry. The number of cell divisions as a measure of cell proliferation was evaluated by using the Proliferation Wizard Module of the ModFit LT Macintosh software. Results are expressed as relative frequency (percentage) of cells undergoing cell divisions. In Figures S3C, S3E, S3H and S3J, the dividing cells are designated by cell division numbers 1, 2, 3, 4, and 5, where number 1 represents parent cell population (blue histogram). In primary glioma culture, there were 38% dividing and 61.7% parent GSCs in IL-17 treatment group (Figure S3E) compared to 34% dividing GSCs and 64% parent in medium control (Figure S3C). Similarly, in U87-MG culture, there were 85% dividing GSCs and 12% parent in IL-17 group (Figure S3I) compared to only 78% dividing GSCs and 20.2% parent in the control (Figure S3H). The data is representative of two independent experiments with similar results.

Supplementary Table S1: Patient profile

Specimen	Final Diagnosis	Age	Sex	Ki-67	GFAP	EGFR
10–040	GBM	59	M	> 70%	+	+
13–033	GBM (oligodendroglial component)	71	M	> 25%	+	strong
13–036	GBM	73	M	30–40%	+	weak
13–037	GBM	62	F	15–20%	+	strong
13–062	GBM	66	F	25%; 90% at foci	variably +	+
13–077	GBM	48	M	scattered	+	N/A
13–102	GBM	63	F	30%	+	+ in infiltrative
14–002	GBM	66	F	8–10%	+	+
14–015	GBM	45	M	25%	+	only focally
SKI 10–1169	GBM	63	M	Scattered 1–2%	+	+
SKI 10–1325	GBM	71	M	focal 15%	strong	strong
SKI 10–3641	GBM (Astrocytoma grade IV)	59	F	up to 50%	strong	strong
